# Postoperative radiograph of the hip arthroplasty: what the radiologist should know

**DOI:** 10.1007/s13244-015-0438-5

**Published:** 2015-10-20

**Authors:** Jan Vanrusselt, Milan Vansevenant, Geert Vanderschueren, Filip Vanhoenacker

**Affiliations:** Department of Radiology, University Hospital Leuven, Gasthuisberg, Herestraat 49, 3000 Leuven, Belgium; Department of Radiology, University Hospital Antwerp, Wilrijkstraat 10, 2650 Edegem, Belgium; Department of Radiology, University of Ghent, De Pintelaan 185, 9000 Ghent, Belgium; Department of Radiology, AZ St-Maarten Duffel/Mechelen, Rooienberg 25, 2570 Duffel, Belgium

**Keywords:** Hip, Arthroplasty, Postoperative complications, Imaging, Radiography

## Abstract

This pictorial review aims to provide the radiologist with simple and systematic guidelines for the radiographic evaluation of a hip prosthesis. Currently, there is a plethora of commercially available arthroplasties, making postoperative analysis not always straightforward. Knowledge of the different types of hip arthroplasty and fixating techniques is a prerequisite for correct imaging interpretation. After identification of the type of arthroplasty, meticulous and systematic analysis of the following parameters on an anteroposterior standing pelvic radiograph should be undertaken: leg length, vertical and horizontal centre of rotation, lateral acetabular inclination, and femoral stem positioning. Additional orthogonal views may be useful to evaluate acetabular anteversion. Complications can be classified in three major groups: periprosthetic lucencies, sclerosis or bone proliferation, and component failure or fracture.

*Teaching Points*

• *To give an overview of the different types of currently used hip arthroplasties.*

• *To provide a simple framework for a systematic approach to postoperative radiographs.*

• *To discuss radiographic findings of the most common complications.*

## Introduction

Hip arthroplasty is one of the most common procedures performed for the treatment of advanced osteoarthritis and is also a required in approximately one-third of hip fracture patients, with 332,000 hip replacements performed in 2010 in the United States [1]. It has been described as one of the most overall successful orthopedic procedures, allowing for early weight bearing and mobilisation, resulting in pain relief, restoration of function, and improved quality of life for many patients [2]. Total hip arthroplasty is most commonly performed for treatment of osteoarthritis. The choice whether to replace a fractured hip with a total hip arthroplasty or a hemiarthroplasty (in which the native acetabulum is spared) remains a topic of an ongoing debate [3]. Since the revolutionary development in the field of hip implants, made by Charnley in the 1960s, surgical techniques and the design of implants as well as the imaging modalities have evolved significantly [4]. Despite the widespread use of MRI, CT, and sonography in joint imaging, the postoperative radiograph remains the keystone in the assessment of hip arthroplasty, as it is readily available at a low cost, with no metal artefact, and facilitating longitudinal comparison. Although cross-sectional studies may have an important role in evaluating and characterizing abnormalities of periprosthetic bone and juxta-articular soft tissues, they usually come at an increased cost. Artefacts still hamper MR image quality and image interpretation, although sequence modification has been shown to allow for evaluation of the bone-prosthesis interface and the surrounding soft tissues. Multidetector CT induces a higher patient radiation exposure compared to conventional radiography. Sonography is not ideally suited to evaluate the prosthesis and periprosthetic bone because of the inability of ultrasound beams to penetrate metal or bone.

## Different types of hip arthroplasty and fixating techniques

### Different types of hip arthroplasty

Basically, hip arthroplasties can be classified into two major types: hemiarthroplasty and total hip arthroplasty.

#### Hemiarthroplasty

**Fig. 1:**
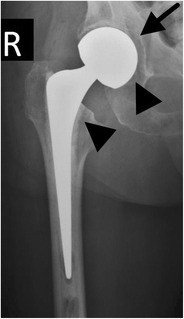
Anteroposterior (AP) radiograph. Cemented unipolar hemiarthroplasty. The femoral stem with the fixed head (*arrowheads*) articulates with the native acetabulum (*arrow*)

**Fig. 2:**
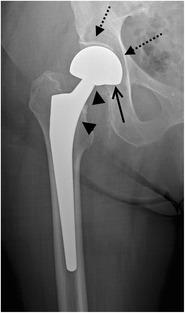
AP radiograph. Cementless bipolar hemiarthroplasty. The femoral stem with a fixed head (*arrowheads*) articulates with a polyethylene lined metal cup (*arrow* indicates position of the radiolucent polyethylene), which articulates with the native acetabulum (*dotted arrow*)

**Fig. 3:**
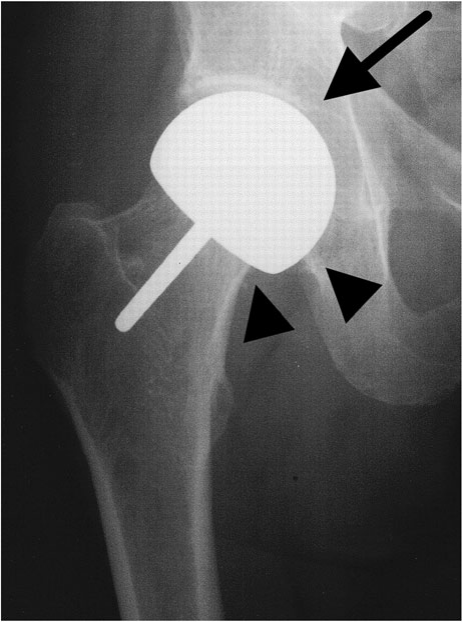
AP radiograph. Cementless resurfacing hemiarthroplasty. Only the femoral head is replaced (*arrowheads*), which articulates with the native acetabulum (*arrow*)

In a hemiarthroplasty, the acetabulum is spared whereas the femoral head and neck are replaced. This type of prosthesis is indicated when the native acetabulum is unaffected. A unipolar hemiarthroplasty consists of a femoral stem with a fixed head, which articulates with the native acetabulum (Fig. 1). A bipolar hemiarthroplasty consists of a femoral stem with a fixed head and a polyethylene lined metal cup, accommodating motion between the cup and the prosthetic head as well as between the cup and the native acetabulum (Fig. 2). In a resurfacing hemiarthroplasty, only the femoral head is replaced (Fig. 3).

#### Total hip arthroplasty

**Fig. 4:**
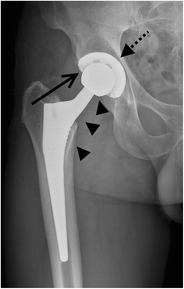
AP radiograph. Cementless total hip arthroplasty. In a total hip arthroplasty, both femoral head and neck (*arrowheads*) as well as the acetabulum (*dotted arrow*) are replaced. The *open arrow* indicates the position of the radiolucent polyethylene cup at the articulation of the prosthetic femoral head and the acetabulum

**Fig. 5:**
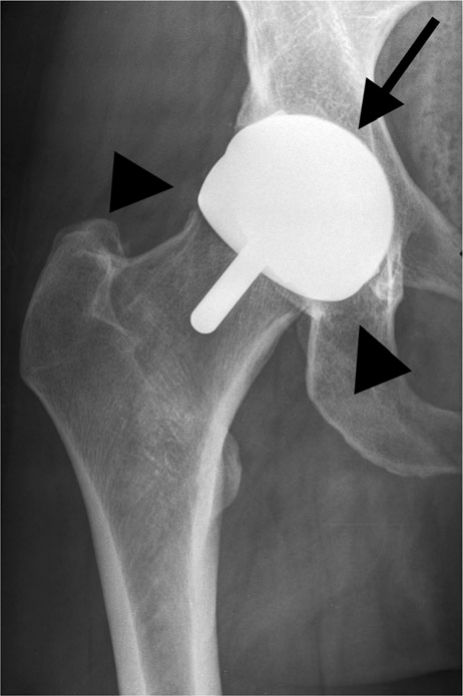
AP radiograph. Cementless resurfacing total hip arthroplasty. In a resurfacing total hip arthroplasty, the femoral head (*arrowheads*) and acetabulum (*arrow*) are replaced. No radiolucent area at the femoral head—acetabulum is noted (metal—on—metal bearing surface)

In a total hip arthroplasty both the femoral head and neck as well as the acetabulum are replaced (Fig. 4). In a resurfacing total hip arthroplasty, the femoral head and acetabulum are replaced, whereas the femoral neck is spared (Fig. 5).

Further subcategorization of hip arthroplasties is based on the identification of which material is used in the bearing surface of the acetabulum and the femoral head. The ‘hard’ bearing surfaces consist of an alloy of metal or ceramic, the ‘soft’ bearing surfaces consist of polyethylene.

### Fixating techniques

In a total hip arthroplasty as in a hemiarthroplasty, a cemented or a cementless stem fixation can be used.

#### Cemented stem fixation

**Fig. 6:**
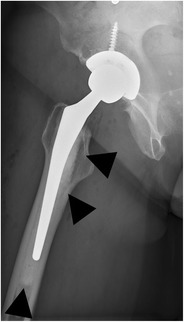
AP radiograph. Cemented total hip arthroplasty, single acetabular screw fixation. In a hybrid cemented arthroplasty, the femoral stem is fixed with cement (*arrowheads*)

**Fig. 7:**
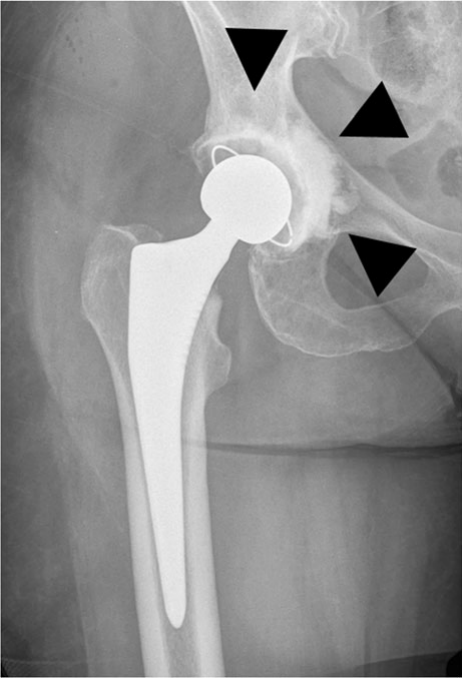
AP radiograph. Cemented total hip arthroplasty. In a reverse hybrid cemented arthroplasty, the acetabular cup is fixed with cement (*arrowheads*)

Bone cement consists of a mixture of an acrylic cement and additives, including Barium (or Zirconium) to render it radio-opaque. Hybrid arthroplasty is a combination of a cemented femoral stem and a cementless acetabular cup, whereas in a reverse hybrid arthroplasty a cementless femoral stem and a cemented acetabular cup are used (Figs. 6 and 7) [5].

#### Cementless stem fixation

**Fig. 8:**
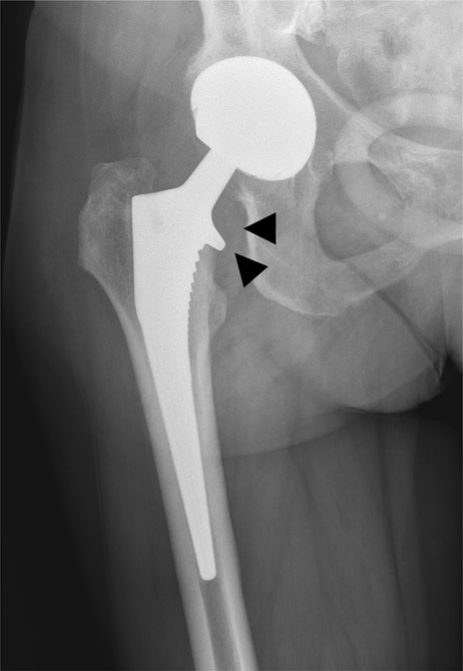
AP radiograph. Cementless total hip arthroplasty. *Arrowheads* indicating the collar of this collared femoral stem

Cementless fixated stems use a press fitting mechanism by placing a slightly oversized stem into a prepared femoral cavity. Its porous coating allows bony ingrowth. These cementless stems exist in a wide variety of forms and shapes, with a collar or without a collar; the stem is in a tapered, anatomical or cylindrical design (Fig. 8). The improved survival of these circumferentially coated uncemented cups and stems has supported their worldwide growing use, despite the higher costs (often approximately three or four times more expensive when compared with the cemented variety).

## Radiographic analysis of a hip arthroplasty

Routine recovery room radiographs are ineffective for screening and unsuitable as baseline for longitudinal follow-up evaluation [6].

Therefore, we recommend a routine, standing anteroposterior (AP) pelvic radiograph, with the hips in extension and maximal internal rotation; the centre of the x-ray beam focused on the pubic symphysis to ensure the inclusion of the entire hip prosthesis and cement [7]. In our institution this standing AP pelvic radiograph is taken at hospital discharge, 6 weeks after surgery and 12 months after surgery, unless pain or clinical symptoms warrants more early investigation.

The assessment of a hip arthroplasty should include the following parameters (Table 1): leg length, vertical and horizontal centre of rotation, lateral acetabular inclination, and femoral stem positioning. The acetabular anteversion is defined on a true lateral radiograph or a cross-table lateral view.

**Table 1:** Parameters to be analysed on each postoperative radiograph after hip arthroplasty

Parameters	Normal findings
Leg length	Acceptable discrepancy of < 1 cm
Horizontal center of rotation	Equal to that of the contralateral hip
Vertical center of rotation	Equal to that of the contralateral hip
Acetabular inclination	Between 30° and 50° (total & resurfacing arthroplasty)
Femoral stem positioning	Neutral alignment with the longitudinal axis of the shaft
Acetabular anteversion (on a lateral radiograph)	Between 5° and 25°
Cement mantle thickness	2–3 mm femoral; no consensus on acetabular mantle thickness (3 mm is suggested)

### Leg length

**Fig. 9:**
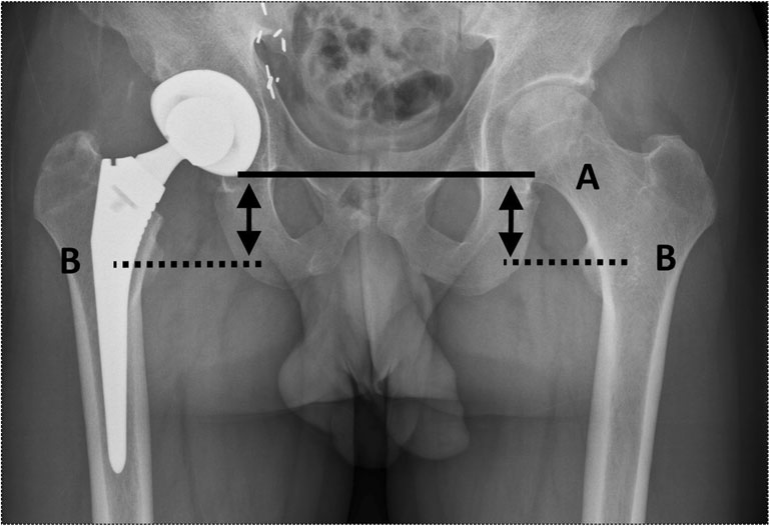
The leg length is measured as the distance between line *A* (connecting the undersurface of the acetabular tear drops) and line *B* (through the middle of the lesser trochanter)

The leg length (Fig. 9) is measured by drawing a line transversely connecting the inferior borders of the acetabular tear drops, the pelvic reference line. The lesser trochanters are used as the femoral reference lines. Perpendicular lines are drawn from the pelvic reference line to the femoral reference lines, the difference between the distances being the leg length discrepancy [8]. Leg length inequality is common after hip arthroplasty; a discrepancy of up to 1 cm is well tolerated. Moderate inequalities are usually corrected with a shoe orthosis.

### The horizontal centre of rotation

**Fig. 10:**
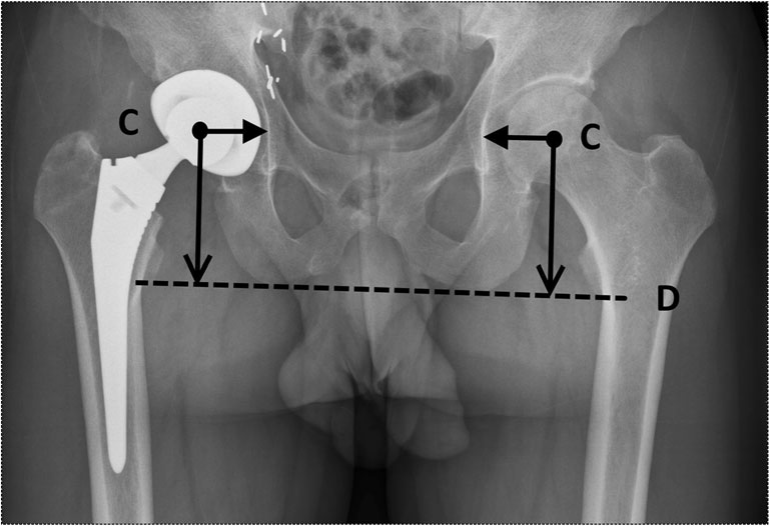
The horizontal centre of rotation is defined as the distance between the centre of the femoral head (point *C*) and the lateral outline of the teardrop shadow. The vertical centre of rotation is defined as the distance between the centre of the femoral head (point *C*) and the transischial tuberosity line (line *D*)

The horizontal centre of rotation (Fig. 10) is defined by the distance between the centre of the femoral head and the teardrop shadow. Ideally, this distance should be equal to that of the contralateral hip; excessive lateral positioning of the acetabular component increases the risk for dislocation and may cause limping.

### The vertical centre of rotation

The vertical centre of rotation (Fig. 10) is defined by the distance between the centre of the femoral head and the transischial tuberosity line. Ideally, this distance should be equal to that of the contralateral hip, mimicking normal anatomy.

### The acetabular inclination

**Fig. 11:**
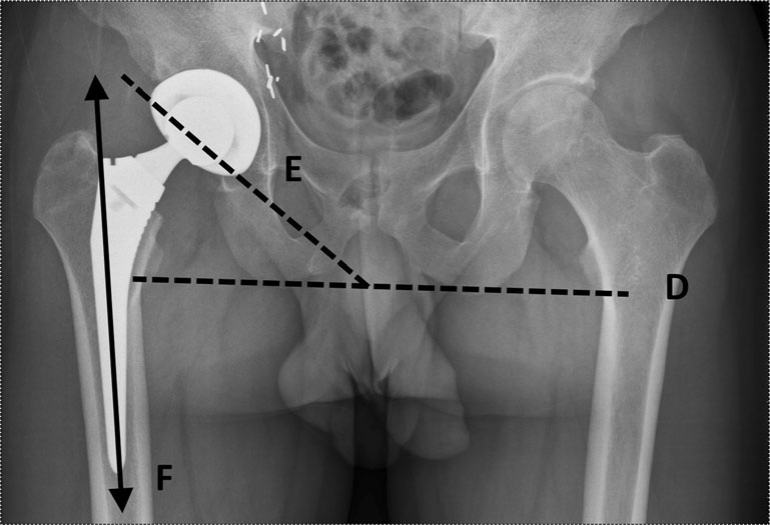
The acetabular inclination is measured by drawing a line through the medial and lateral margins of the cup (line *E*) and measuring the angle with the transverse pelvic axis (line *D*). The femoral stem positioning should be aligned with the longitudinal axis of the shaft (line *F* = normal, longitudinal axis of the shaft)

The acetabular inclination (Fig. 11) is the angle between the articular side of the acetabular cup and the transverse axis. Measurement of this angle can be done by drawing a line through the medial and lateral margins of the cup and measuring the angle with the transischial tuberosity line. The normal range of inclination is between 30 and 50° [9]. Smaller angles provide a stable hip but a reduced abduction. Greater angles are associated with greater risk of hip dislocation.

### Femoral stem positioning

**Fig. 12:**
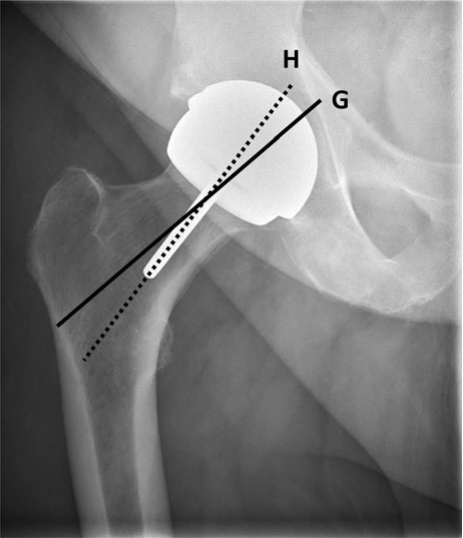
Normal valgus positioning of the femoral stem in a resurfacing arthroplasty (line *H*) compared with the longitudinal axis of the femoral neck (line *G*)

Ideally, the position of the femoral stem (Fig. 11) on an AP view should be seen in neutral alignment with the longitudinal axis of the femoral shaft, and the tip situated in the centre of the shaft. Many studies have shown that failure of the femoral stem is associated with varus malpositioning [10–12]. The femoral component of a resurfacing arthroplasty should be placed in a relative valgus position of 5°–10° (Fig. 12).

### The acetabular anteversion

**Fig. 13:**
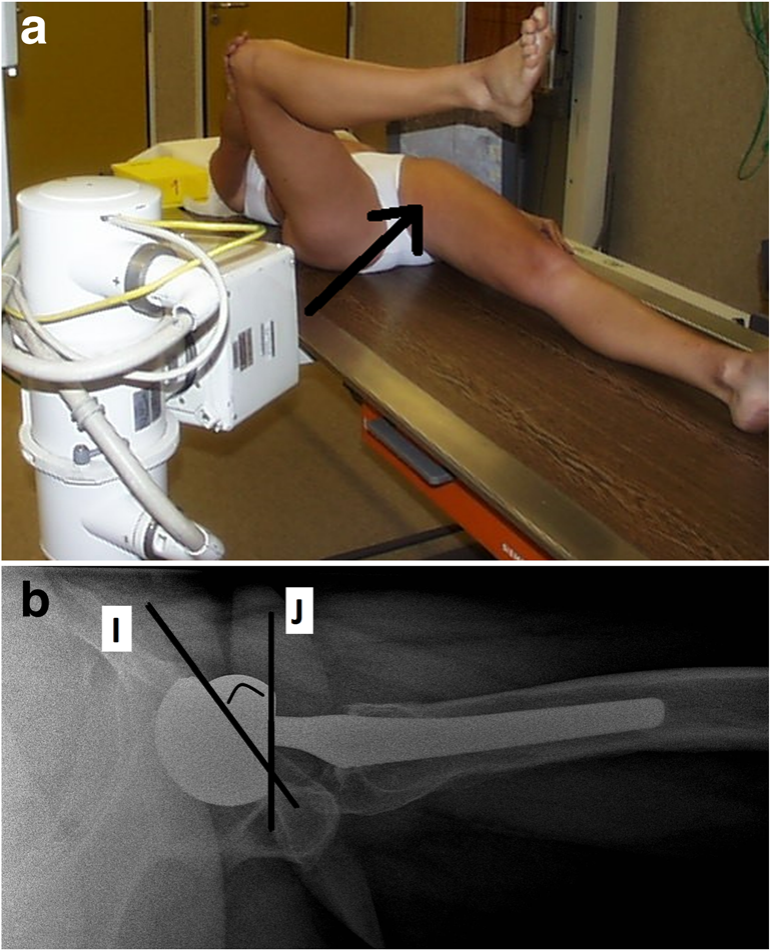
**a** Photograph of the patient positioning for a lateral view of the hip, the *arrow* indicating the direction of the x-rays. **b** Lateral radiograph. The acetabular anteversion is defined by the angle between the acetabular axis (line *I*) and the coronal plane (line *J*). In this patient, the angle measures approximately 25° (normal range between 5°–25°)

The acetabular anteversion is defined on a lateral view by the angle between the acetabular axis and the coronal plane (Fig. 13). Normal value ranges from 5° to 25° anteversion as this allows adequate flexion of the hip [13]. Acetabular retroversion predisposes to hip dislocation.

### The cement mantle

The cement-bone interface, the cement-prosthesis interface and the cement thickness should be scrutinized for the presence of any gaps or lucencies. There is no consensus –however- on the ideal acetabular cement mantle thickness in vivo (in vitro evaluation suggested an optimal thickness of 3 mm) [14]. Complete femoral cement mantles of 2–3 mm have been shown to yield good long term outcome [15].

**Fig. 14:**
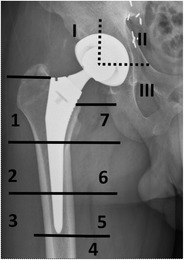
Standardized template for radiographic assessment of periprosthetic lucency, with three acetabular zones (*I*–*III*) and seven femoral zones (*1*–*7*)

For localization of cement-related or periprosthetic abnormalities at the acetabular and femoral components, standardized templates have been described by Charnley-Delee [16] and Gruen [17], respectively (Fig. 14).

## Most common complications

The radiographic features relating to complications or failure can be classified into three major categories, based on their radiographical appearance.

### Periprosthetic lucencies

**Fig. 15:**
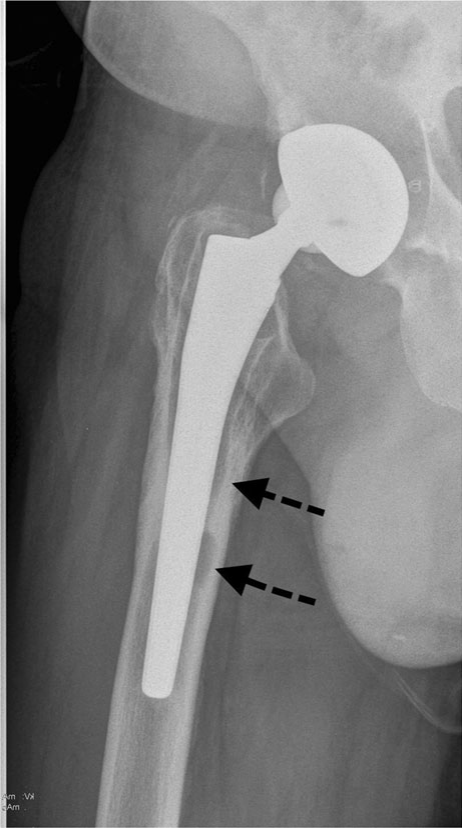
AP radiograph, 3 years postoperatively. Cementless bipolar hemiarthroplasty. Aseptic loosening, radiographically seen as periprosthetic lucencies in a Gruen zone 5/6 (*arrows*)

*Aseptic loosening or osteolysis* (Fig. 15) is a biological process that is initiated by macrophage phagocytosis of particulate debris, causing an aseptic foreign body granulomatosis [18]. The implant becomes separated from the host bone, resulting in mechanical (aseptic) loosening. On a radiograph, this manifests as a periprosthetic zone of radiolucency around the bone-cement or the bone-prosthesis interface.

**Fig. 16:**
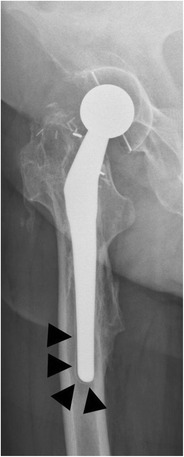
AP radiograph. Cementless total hip arthroplasty. A < 2 mm lucency, outlined by a discrete sclerotic margin, in Gruen zone 3/4 (*arrowheads*): this indicates a fibrous rather than bony ingrowth, thought to provide sufficient stability

Aseptic loosening and osteolysis should be differentiated from other, nonpathological causes of periprosthetic lucencies. In a cemented arthroplasty, a < 2 mm lucency at the bone-cement interface indicates the formation of a fibrous membrane (representing the lucency), outlined by a thin, sclerotic demarcation line [19]. This is thought to represent a stable fibrous reaction to cement. In a cementless arthroplasty, a similar < 2 mm lucency also outlined by a thin sclerotic line, along a polished segment where no bony ingrowth is expected, indicates fibrous bony ingrowth and is thought to provide sufficient stability (Fig. 16) [20].

As a rule of thumb, periprosthetic lucencies wider than 2 mm and/or progressive lucencies are signs of abnormality.

**Fig. 17:**
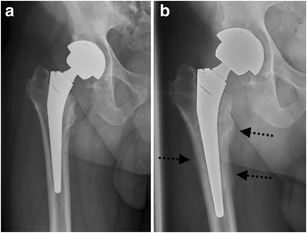
**a** AP radiograph, 1 month postoperatively. Cementless total hip arthroplasty. Normal postoperative findings. **b** AP radiograph of the same patient, 3 months postoperatively. Periosteal reaction in Gruen zone 2/5/6/7 (*arrows*): proven case of infection

**Fig. 18:**
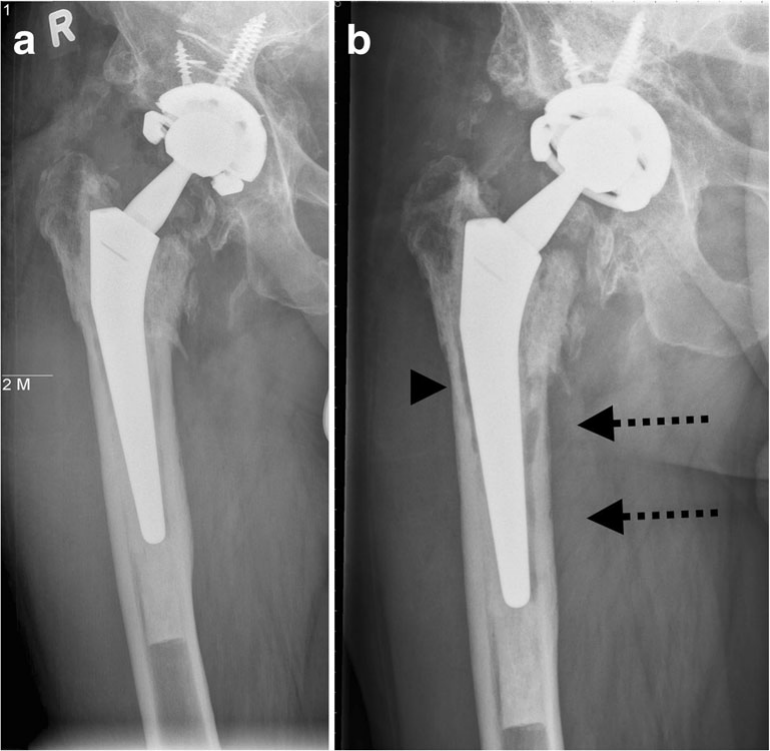
**a** AP radiograph, 4 months postoperatively. Cemented (hybrid) total hip arthroplasty after revision with acetabular fixation screws. Normal postoperative findings. **b** AP radiograph of the same patient, 5 months postoperatively. Periprosthetic lucencies in Gruen zone 5/6 (*arrows*) and more discrete in Gruen zone 2 (*arrowhead*): proven case of infection

*Infection* remains a major and devastating long-term complication, occurring in 1–2 % [21]. Similarly to aseptic loosening, plain radiography shows a periprosthetic zone of radiolucency around the bone-cement or the bone-prosthesis interface. The differential diagnosis between septic and aseptic loosening can be very challenging, especially when no previous radiographs are available. However, the presence of a femoral periosteal reaction [22] (Fig. 17) or rapid progressive disease [23] (Fig. 18) are indicative of septic rather than aseptic loosening.

**Fig. 19:**
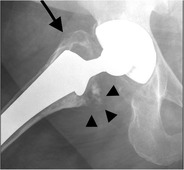
AP radiograph. Cementless total hip arthroplasty. Periprosthetic lucency in the area of the greater trochanter (*arrow*) and some punctate densities adjacent to the lesser trochanter/ Gruen zone 7 (*arrowheads*), representing small metal particles: adverse reaction to metal debris

Deposition of metallic wear particles in periprosthetic tissues (Fig. 19) may occur, particularly in metal-on-metal bearing arthroplasty. This process has been given the umbrella term *‘adverse reaction to metal debris’*, including metallosis [24], aseptic lymphocytic vasculitis associated lesions [25] and pseudotumours [26]. Radiographs usually show normal findings, but in longstanding cases there may be evidence of loosening or, in a resurfacing arthroplasty, pressure erosion on cortical bone [27].

### Sclerosis and bone proliferation

**Fig. 20:**
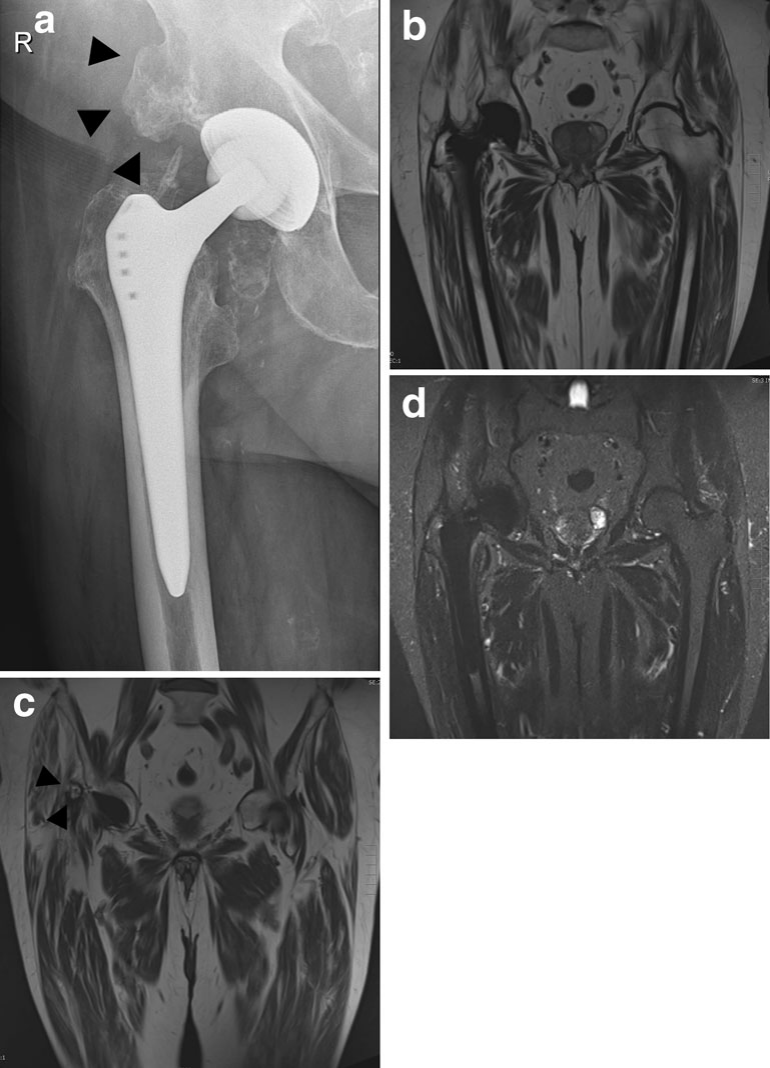
Although current state-of-the art MRI with Metal Artefact Reduction Sequences allows assessment of correct position of the hip prosthesis as well as periarticular abnormalities, mature heterotopic bone formation (*arrowheads* in *A* and *C*) is often more readily visible on plain radiographs than on MRI due to similar signal of mature bone marrow and fatty infiltration within the gluteus musculature at the site of the hip prothesis. **a** AP radiograph. Cementless total hip arthroplasty. Heterotopic bone formation (*arrowheads*), 7 years postoperatively. **b** T1-weighted, coronal image (WI) of the pelvis in the same patient. **c** T1-weighted, coronal image (WI) of the pelvis at a more anterior location barely showing heterotopic bone formation (*arrowheads*). **d** STIR, coronal image of the pelvis in the same patient

**Fig. 21:**
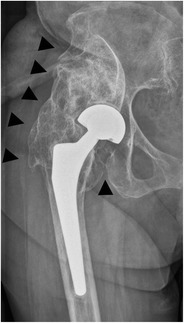
AP radiograph. Cementless bipolar hemiarthroplasty. Extensive heterotopic bone formation (*arrowheads*), bridging from femur to pelvis, restricting abduction

Development of bone outside its normal location in the skeleton is termed *heterotopic bone* formation, occurring in up to half of patients; this rarely results in significant limitation of movement (Figs. 20 and 21) [28].

**Fig. 22:**
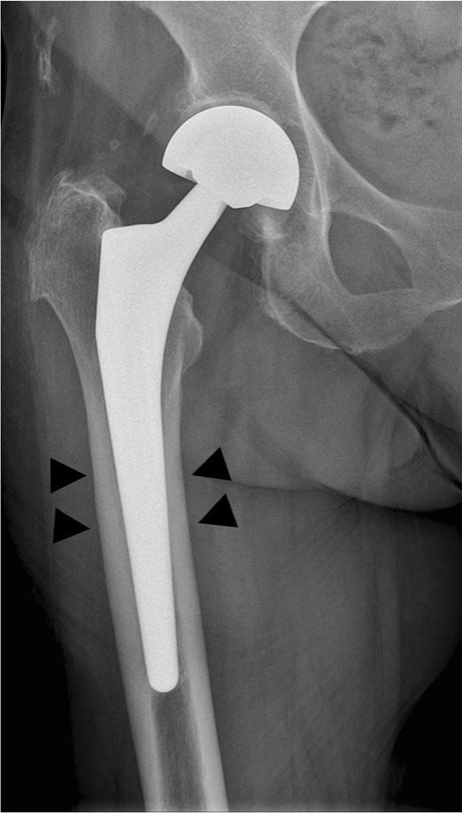
AP radiograph. Cementless bipolar hemiarthroplasty. Spot welding (new bone formation originating from the endosteal surface and reaching the prosthesis) in Gruen zone 2/6 (*arrowheads*)

*Spot welding* consists of new bone formation originating from the endosteal surface and reaching the prosthesis. This is mostly seen in cementless femoral stems and is a strong indicator of stability (Fig. 22) [29].

**Fig. 23:**
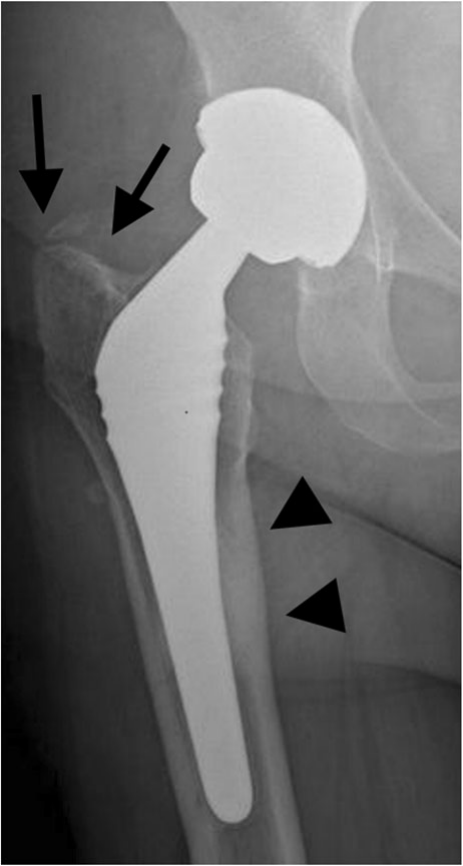
AP radiograph. Cementless total hip arthroplasty. Stress shielding. Cortical hypertrophy in Gruen zone 1 (*arrowheads*) and adaptive atrophy in Gruen zone 6 (*arrows*) as different parts of reactive bone remodelling

*Stress shielding* refers to the transfer of the normal load from the femoral neck and intertrochanteric region to the proximal femoral diaphysis (the hip implant causes altered mechanical forces), causing bone resorption on the lateral side of the proximal femur, most commonly seen in Gruen zone 1, as well as bone hypertrophy at the medial side of the proximal femur (Fig. 23). This process implies stability and should not be misinterpreted as a complication [30].

**Fig. 24:**
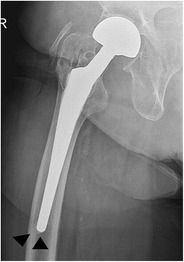
AP radiograph. Cementless bipolar hemiarthroplasty. Bone pedestal in zone 4 (*arrowheads*). The association with loosening remains unclear

Sclerosis at the tip of a cementless femoral component, bridging the medullary canal, is a *bone pedestal* (Fig. 24). The association of this often incidentally found entity with loosening remains unclear [31].

### Component failure/ fracture

**Fig. 25:**
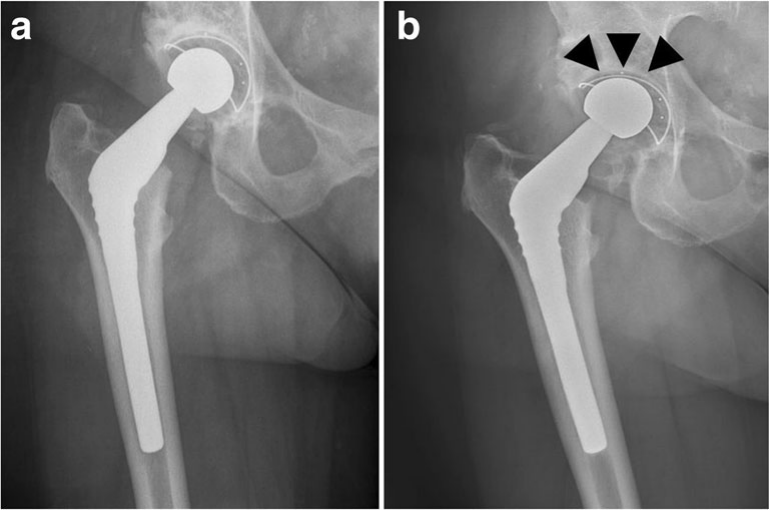
**a** AP radiograph, 6 months postoperatively. Cementless total hip arthroplasty. Normal postoperative findings. **b** AP radiograph of the same patient, 4 years postoperatively. Cranial displacement of the femoral head in the acetabular cup (*arrowheads*), indicating linear wear

*Linear wear* occurs typically in hip arthroplasty with a polyethylene component (hard-on-soft or soft-on-soft bearing surface combination). An asymmetric position of the femoral head within the acetabular cup on radiographs is a definite sign of polyethylene wear (Fig. 25).

**Fig. 26:**
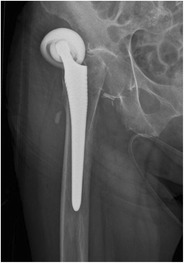
AP radiograph. Cementless bipolar hemiarthroplasty. Lateral dislocation of head and acetabular cup

The reported rate of *dislocation* varies from 0.5 to 10 % after primary total hip arthroplasty [32]. Most dislocations occur in the early postoperative period, during the initial weight bearing (Fig. 26) [33]. Abnormal acetabular inclination, acetabular retroversion or an incorrect center of rotation, among others, increase the likelihood of dislocation.

**Fig. 27:**
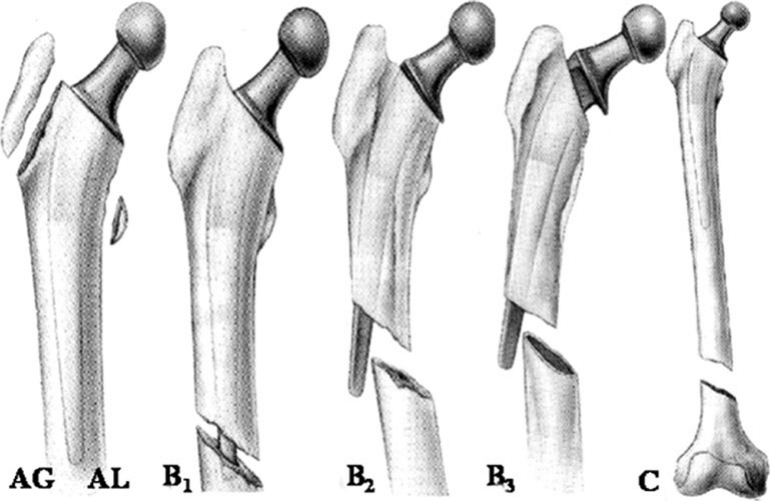
Vancouver classification of periprosthetic fractures. *Type A* fractures are peritrochanteric fractures (subtypes *AG*: greater trochanter and *AL*: lesser trochanter). *Type B* fractures occur around or just below the tip of the femoral stem (subtypes *B1*: stable stem, *B2*: loose stem, *B3*: loose implant with substantial bone loss). *Type C* fractures occur well below the implant (image courtesy of Hwang KT, Kim YH (2011) Treatment of periprosthetic femoral fractures after hip arthroplasty. J Korean Fract Soc 24:121–130)

**Fig. 28:**
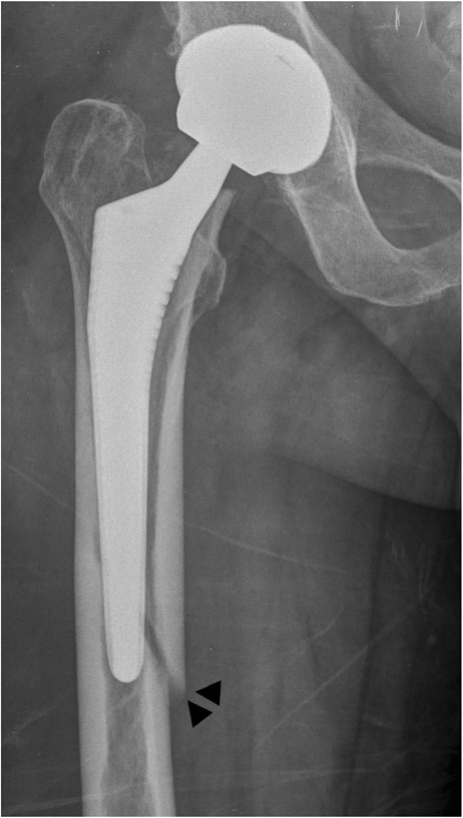
AP radiograph. Cementless total hip arthroplasty. Periprosthetic fracture in Gruen zone 5 (*arrowheads*), Vancouver type B1 fracture

*Periprosthetic fractures* occur more often around the femoral than the acetabular component, be it intra- or postoperative. The Vancouver classification divides the periprosthetic, postoperative fractures of the femur into three major types (Fig. 27) [34]. Postoperative femoral fractures occur typically, but not exclusively, at the level of the tip of the femoral stem (Fig. 28).

**Fig. 29:**
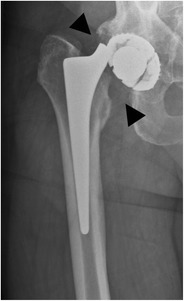
AP radiograph. Cementless total hip arthroplasty. Proximal prosthetic/metallic fracture through the neck of the femoral implant (*arrowheads*)

*Prosthetic fractures* occur mostly in the femoral stem of the implant, representing a metal-fatigue stress fracture; this typically occurs in prostheses that are well fixed distally but are mobile proximally and result in fractures through the middle or proximal third of the stem (Fig. 29) [35]. Patients with increased body mass index (BMI) are at greater risk of reaching an implant failure point due to fatigue loading [36]. Varus malpositioning predisposes to fractures of the femoral stem.

## Conclusion

Despite the widespread and growing use of MRI, CT, and sonography in imaging the joint, the postoperative radiograph is still the mainstay in assessing postoperative hip arthroplasty and its follow-up. Serial radiography is often the most useful imaging method to detect, sometimes subtle, complications.

We recommend using a standardized radiological approach in assessing the postoperative radiograph of a hip arthroplasty, at hospital discharge, 6 weeks after surgery and 12 months after surgery, unless pain or clinical symptoms warrants more early investigation.
